# Individual Differences in Holistic and Compositional Language Processing

**DOI:** 10.5334/joc.283

**Published:** 2023-06-28

**Authors:** Kyla McConnell

**Affiliations:** 1 English Department, Albert-Ludwigs-Universitat Freiburg, Freiburg im Breisgau, DE

**Keywords:** backward transition probability, individual differences, chunking, inhibition, shifting, lexical co-occurrence

## Abstract

Individual differences in cognitive abilities are ubiquitous across the spectrum of proficient language users. Although speakers differ with regard to their memory capacity, ability for inhibiting distraction, and ability to shift between different processing levels, comprehension is generally successful. However, this does not mean it is identical across individuals; listeners and readers may rely on different processing strategies to exploit distributional information in the service of efficient understanding. In the following psycholinguistic reading experiment, we investigate potential sources of individual differences in the processing of co-occurring words. Participants read modifier-noun bigrams like *absolute silence* in a self-paced reading task. Backward transition probability (BTP) between the two lexemes was used to quantify the prominence of the bigram as a whole in comparison to the frequency of its parts. Of five individual difference measures (processing speed, verbal working memory, cognitive inhibition, global-local scope shifting, and personality), two proved to be significantly associated with the effect of BTP on reading times. Participants who could inhibit a distracting global environment in order to more efficiently retrieve a single part and those that preferred the local level in the shifting task showed greater effects of the co-occurrence probability of the parts. We conclude that some participants are more likely to retrieve bigrams via their parts and their co-occurrence statistics whereas others more readily retrieve the two words together as a single chunked unit.

## 1. Introduction

There is vast cognitive diversity amongst proficient native speakers of any given language. Speakers have differing underlying capacities for memory, resistance to distraction, and ability to focus; they have their own processing strategies, preferences, and personality traits. These underlying traits and abilities compound on lifetimes of unique language experience in different contexts and with different interlocutors. Linguistic research increasingly accepts that language processing varies as a function of both an individual’s inherent traits as well as their experience throughout the lifespan. However, as long-held assumptions of a uniform, idealized speaker/hearer are replaced by theories that allow for considerable individual differences, the axes along which speakers of the same language vary are called into question.

In the following paper, we focus specifically on five primarily inherent sources of potential individual differences: cognitive inhibition, global/local shifting ability, verbal working memory, personality, and processing speed. These build on previously-established experienced-based individual differences in probabilistic language processing (McConnell & Blumenthal-Dramé, 2021). To this end, we captured response times (RTs) from self-paced reading of naturalistic bigrams (e.g., “apparent conflict”), calculated their association strength via corpus-extracted backward transition probability (BTP) and modeled the relationship between probabilistic language processing and the selected cognitive abilities, capacities, and preferences.

### 1.1 Backward transition probability

As they move through the world, both proficient adult speakers and young L1 learners pay attention to the distributions inherent in the language around them (Aslin, 2017). Through a process of statistical learning, language users keep track of the frequency with which certain syllables, morphemes, and words co-occur. Sensitivity to co-occurrence is a key component of native language acquisition; learners break down acquired multiword units (e.g., “gimme-it”), which are initially un-analyzed chunks, into their component parts (i.e., “give” + “me” + “it”) (Abbot-Smith & Tomasello, 2006; Arnon, 2009; Lieven et al., 2009). Usage-based and emergentist theories of linguistics posit that this sensitivity continues throughout a speaker’s life, with each exposure to language leaving a memory trace that updates their mental representation. This results in implicit knowledge of the frequency of not only single words, but also multi-word units (Arnon & Snider, 2010; Behrens & Pfänder, 2016; Carrol & Conklin, 2020; Divjak, 2019; Gries & Divjak, 2012).

Which units are then stored as wholes, and which are constructed from their parts? It appears that units are stored and retrieved redundantly at both the level of the “part” and the “whole” (Blumenthal-Dramé, 2017; Bybee, 2010; Hilpert, 2014; Yang et al., 2020). That is, smaller, decomposed units (e.g. morphemes, words) exist along a spectrum of relative cognitive prominence along with their complex forms (e.g. multi-morphemic words, multi-word units) (Hay & Baayen, 2002). A continuum emerges in terms of how accessible the representation of the whole is relative to the representation of the part (Hay & Baayen, 2005), and this continuum is likely affected by both participant-level and item-level variables. One item-level variable may be the relative frequency of parts to wholes (i.e., “worthless” is more frequent than “worth” so is often processed at the chunk level, but “tearless” is less frequent than “tear”, so it is more likely to be constructed compositionally) (Blumenthal-Dramé, 2017; Blumenthal-Dramé et al., 2017; Hay & Baayen, 2005). Additional factors are also known to affect chunk status both within and outside of the linguistic domain, including expertise, compositionality, and salience, among others (Gobet et al., 2016; Gobet & Clarkson, 2004; A. Goldberg & Suttle, 2010; Miller, 1956; Yang et al., 2022).

In the current experiment, we focus on the frequency-based relationships between words, and take a common metric of lexical co-occurrence to represent the relative frequency of parts to wholes: backward transition probability (BTP). This simple interlexical statistical metric measures the frequencies of one individual unit compared to the frequency of its co-occurrence in a larger whole; specifically, the likelihood of part two being directly preceded by part one. In the scope of the current paper, BTP is calculated as the likelihood of word 1 (W1, e.g., *absolute*) directly preceding word 2 (W2, e.g., *silence*), compared to all instances of W2, though the metric is not limited to lexical units and can be calculated at any grain size. For example, if *absolute silence* has a corpus frequency of 154, and *silence* appears 36,701 times as a noun in the same corpus, the bigram is given a BTP score of 154/36701 (approximately .004).

BTP between words (and particularly, in bigrams) has been found to affect reading times and eye movement behavior when reading (e.g., Demberg & Keller, 2008; Kapteijns & Hintz, 2021). The metric has been used to operationalize diverse theoretical concepts in linguistics, including probabilistic integration of unfolding words or phrases into previously-encountered contexts and structures (also called “retrodiction”) (Ferreira & Chantavarin, 2018; Onnis & Huettig, 2021; van Paridon & Alday, 2020), and sensitivity to phonotactic contingencies and lexical co-occurrence (McConnell & Blumenthal-Dramé, 2019; Perruchet & Desaulty, 2008). The measure has also been used to model language learning processes (McCauley & Christiansen, 2019; Pelucchi et al., 2009; Roete et al., 2020). BTP’s sibling metric, forward transition probability (FTP), has also received wide-spread attention, along with its log-inverse: “surprisal” (Boston et al., 2008; Frisson et al., 2005; Hale, 2016; Levy, 2008; Lowder et al., 2018; McDonald & Shillcock, 2003; Smith & Levy, 2013; Willems et al., 2016). Although the two metrics are similar, they have been found to be independently informative of real-time language processing (Onnis & Huettig, 2021).

It is reasonable to assume that BTP could be a predictive metric for sentence reading in general, i.e., at any point in a given sentence. Modifier-noun bigrams, however, are a particularly interesting stage upon which to observe this phenomenon for multiple reasons: First, they are easily extracted from corpora. At the same time, grammatical structure is kept constant (thereby avoiding potential influence from confounds like syntactic structures and parts of speech). There are also observable probabilistic preferences for one modifier over another even when there are multiple synonymous options (e.g., ‘vast majority’ vs. ‘large majority’). Additionally, modifier-noun NPs are right-headed in English, i.e., the head noun appears after or to the right of potential modifiers. Because the head noun is both obligatory and carries the main semantic weight of the phrase, orienting towards this important lexeme may take primacy over pre-empting potential modifiers. This is supported by previous research, which shows that BTP is a stronger predictor of reading times for modifier-noun bigrams than FTP and other corpus linguistic association measures (McConnell & Blumenthal-Dramé, 2019, 2021), particularly for speakers of English, as compared to a left-branching language like Korean (Onnis & Thiessen, 2013).

Therefore, we take BTP to operationalize the prominence of the wholes to the parts in the modifier-noun bigrams that make up the stimuli in this experiment. Specifically, we measure the BTP score between the modifier and the noun in bigrams like “absolute silence” (see Section 2.1 for more about stimuli). We assume that BTP represents the relative prominence of the chunk (the bigram) compared to its component parts (the individual words) because it is calculated via the frequencies of the bigram and the frequency of the noun. We hypothesized that comprehenders would emerge on a spectrum between a preference for the chunk level and a preference for a rather atomistic or compositional approach; this hypothesis is in line with theories of visual perception, which have found that factors like age, expectation, and even ability for creative thinking can affect global precedence biases (Behrmann et al., 2014; Lee et al., 2021; Staudinger et al., 2011; Summerfield & Egner, 2009; Zabelina & Ganis, 2018). This difference should be apparent in (self-paced) reading times to the noun in the bigram, as it is the point in which the comprehender can process and integrate the chunk (i.e., the bigram). Further, we postulated that there would be identifiable individual differences that modulate which processing style a particular comprehender prefers (McCauley & Christiansen, 2015).

### 1.2 Endogenous individual differences

Usage-based theories have widely postulated and collected evidence for the influence of experience, including age, years of formal education, print exposure, and even the kinds of texts readers have been exposed to (Dąbrowska, 2018, 2019; Verhagen et al., 2018). Age and reading experience have been established as important modulators of reading strategy in the context of modifier-noun compounds specifically; older readers and those with less reading experience, i.e., those readers who may have greater need for compensation, showed stronger effects of transition probability (McConnell & Blumenthal-Dramé, 2021). However, these experience-based, or “exogenous”, factors are not the sole determiners of individual differences; there are also a host of “endogenous”, i.e., inherent, factors that seem to play a role in language processing (Kerz & Wiechmann, 2021; Kidd et al., 2018; Kidd & Donnelly, 2020; Ryskin et al., 2020).

In the current experiment, we account for the known experience-based predictors of age, education, and reading experience while investigating the explanatory potential of five endogenous cognitive abilities that might be related to processing style. Of course, creating a binary distinction between endogenous and exogenous traits is a simplification; many (if not most) endogenous traits are likely affected by experience. However, this distinction highlights that the factors we focus on here are not primarily experience based. Specifically, the current experiment investigates two measures (global/local shifting and inhibition) that reflect processing preferences for the holistic or the atomistic level and two cognitive abilities known to affect language comprehension (verbal working memory and reaction speed). The fifth factor, personality, is an exploratory variable that has newly been gaining ground in linguistic research for its ability to capture underlying processing preferences and styles that may account for achievement in relevant abilities like statistical learning.

#### 1.2.1 Cognitive inhibition

The first ability that we hypothesized to be relevant to the access and processing of multiword units was cognitive inhibition, the ability to resist distracting stimuli or suppress dominant reactions (Butterfuss & Kendeou, 2018; Friedman & Miyake, 2004a). There are several assessments of cognitive inhibition that tap into different aspects of the ability, specifically: resistance to interference from distractors, inhibition of prepotent responses or inhibition of previously necessary information that is no longer relevant (Friedman & Miyake, 2004a). We selected an Eriksen flanker task, which is correlated with the first aspect – the ability to focus attention on a necessary item and inhibit the rest. This type of inhibition “has been associated with focused attention or selective enhancement for target stimuli”; the task also includes looking at a whole visual array and being able to selectively focus on a particular part (the middle item) (Friedman & Miyake, 2004a, p. 105).

For this task, participants were asked to focus on the middle figure in a series of five figures (arrows, in this case) and to press a key corresponding to the direction of that figure.^1^ The task tends to have low task difficulty in average populations (Zirnstein et al., 2018), potentially because it is possible to adopt a purely vision-based strategy if the position of the critical stimulus is always in the same spot; to combat this, we jittered the position of the stimulus very slightly and removed the fixation cross, thereby increasing the likelihood that a participant had to see the entire stimulus including the distracting flanks. The task included 50 trials and 5 practice items.

Inhibition is widely acknowledged to play a crucial role in language processing, although the details have not been fully resolved. Successful comprehension requires the inhibition of unintended but possible interpretations of anaphors, homophones and homonyms (Gernsbacher, 1993; Gernsbacher & Robertson, 1995; Myers & O’Brien, 1998). It has also been suggested that proficient readers are more able to focus on task-related goals, with less intrusions of unrelated content (De Beni & Palladino, 2000; Gernsbacher, 1993). Working memory may also be overloaded in individuals with lower inhibitory control, because possible but improbable interpretations may not be inhibited quickly or efficiently enough for optimal online language comprehension (Butterfuss & Kendeou, 2018; Cain, 2006; De Beni & Palladino, 2000). In the current setting, inhibition is operationalized as an inhibition of the global environment, an ability that could be relevant to a reader’s tendency to process in a holistic or compositional way.

Given the task setup, the Flanker task asks participants to inhibit distractions in the global environment and focus on the critical part. In the matched condition, the part and the whole do not compete, but in the mismatched condition, the participant must direct their attention to the individual units rather than drawing meaning from the larger unit. If reaction times are significantly larger in the competing condition (i.e., the participant has a high Flanker score), this could indicate a comparatively greater difficulty inhibiting at the chunk level, perhaps because the chunk level is strongly activated or preferred. We thus hypothesized that individuals who processed more holistically would receive higher Flanker scores.

#### 1.2.2 Global/local shifting

Shifting, a domain-general ability for directing attention to different levels or sources of information (Miyake & Friedman, 2012), was also hypothesized to be relevant to attending to language input at the level of the whole and/or the level of the part. Just as reading requires shifting between the phonological and orthographic levels and the semantic and discourse levels (Butterfuss & Kendeou, 2018), processing bigrams likely also requires shifting between the level of the part (the individual words) and the level of the whole (the bigram).

Not only do individuals differ in their ability to shift between different levels, but they also may have a default preference for processing at a certain level (which may be a gradient phenomenon ranging in intensity). Indeed, although most healthy adults recognize the holistic level before the local level, many factors influence this preference including age, experience, psychopathology and culture (Bellgrove et al., 2003; Chamberlain et al., 2017; Chua & Gauthier, 2020; Lao et al., 2013; Lewis & Dawkins, 2015; Staudinger et al., 2011; Van der Hallen et al., 2015). We expect an interaction between preference for the global or local level and the ability to shift between them; individuals with strong shifting ability will show a weaker preference to their ‘default’ or preferred level compared to those with weaker shifting ability.

One task that taps into global/local shifting ability and preference is the Navon task, in which participants are directed to focus their attention on (and respond based on) either a large (global) shape or its (local) component shapes based on a by-trial cue. In this way, they are asked to switch attention between the “forest” and the “trees”. We implemented a classic ‘Navon’ global-local scope test, in which participants saw many small shapes of the same type (i.e., many small circles) that together made up the image of a larger shape (i.e., in the shape of an X). Depending on the color of the shapes, they were asked to attend to just one of these constructs (the smaller or the larger shapes) and respond according to that level by identifying the shape. In doing so, they were required to shift attention between the global level and the local level on a task-by-task basis (Navon, 1969). The task included 50 trials and 10 practice items. The final score was calculated as the difference between the average time (in milliseconds) for a response to the local condition subtracted from the average time of response in the global condition.

We interpret the results of a Navon task as revealing not just an inherent preference for either the “global” or the “local” level but also the ability to dynamically shift between levels of representation as needed. Scores around 0 indicate that RTs to the global condition were similar to RTs in the local condition. This could reflect an ability to shift dynamically between the global and the local level or at least a similar amount of difficulty in the global as in the local condition. However, deviations in either direction (scores larger or smaller than 0) indicate that one of the two conditions is processed more efficiently. If an individual scores high on the Navon task because they have an inherent preference for the holistic level, they may be quick to abandon a compositional reliance on BTP as the prevalence or frequency of the bigram increases. Those who score lower, on the other hand, may be inherently atomistic and thus necessarily compositional.

#### 1.2.3 Verbal working memory

Working memory capacity has long been a candidate for a cognitive ability that is correlated with language comprehension in both adults and children (Daneman & Carpenter, 1980; Kidd et al., 2018; MacDonald & Christiansen, 2002). This ability, which involves storing and manipulating a limited amount of information for a short time, can be further sub-divided in verbal and non-verbal working memory (Baddeley, 1992, 2012). Readers with greater verbal working memory abilities have been found to more efficiently understand syntactically rare constructions like “garden path” sentences and object relatives, potentially because they are able to keep more than one possible sentence completion activated in memory while processing (Daneman & Carpenter, 1980; Just & Carpenter, 1992; King & Just, 1991; MacDonald et al., 1992; Wlotko & Federmeier, 2012).

Working memory is closely tied to long-term memory, serving as the workbench in which current stimuli can be tied to stored knowledge from previous experience (Huettig & Janse, 2016; Slevc & Novick, 2013). In terms of reading modifier-noun bigrams, this ability could serve as the nexus between incoming linguistic input and distributional statistics such as BTP, which are likely to be stored in long term memory. Working memory also affects implicit sequence learning in the visuomotor domain, with higher WM participants found to chunk at larger grain sizes (Medimorec et al., 2019). For these reasons, we expected an effect of BTP but were agnostic as to the direction: either higher WM could lead to an increased reliance on BTP and related distributional information that is stored in long term memory, or higher WM could allow for larger chunk sizes and thus index holistic processing.

Participants in the current experiment completed a classical verbal working memory task: the reading span task (abbrv. RST) (Daneman & Carpenter, 1980; Friedman & Miyake, 2004b; Klaus & Schriefers, 2016; Redick et al., 2012). For this, two tasks are interspersed: participants are (A) shown a single word which they are instructed to hold in memory and then (B) asked to read sentences and decide if they “make sense”, which serves as a language-related distractor interfering with the storage of language input. These two components are repeated 3 to 6 times, forming a set, and at the end of the set, participants must recall the words that were presented in task A. The task included 11 sets (54 total sentences) and 3 practice sets (9 sentences).

#### 1.2.4 Personality traits

**Figure 1:**
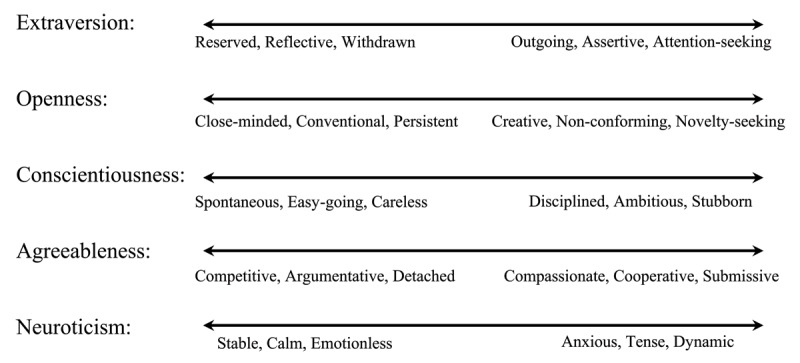
The personality continuums measured by the Big 5.

Recently, research has become increasingly interested in a potential new source of individual difference in language skills: personality traits. Personality is not only a socio-cultural construct but has also been deconstructed into five core cognitive tendencies in gathering, processing and sharing information (Goldberg, 1990; McCrae & John, 1992; Wiggins, 1996). The “Big Five” personality traits measure extraversion, openness, conscientiousness, agreeableness and neuroticism (also called emotional stability) (see Figure 1) and they have been found to correlate with the volume of brain regions associated with similar personality characteristics (DeYoung et al., 2010). The Big Five traits have been associated with differences in academic achievement and L2 learning (Grey et al., 2015; Komarraju et al., 2011; MacIntyre & Charos, 1996), and recently, in sensitivity to linguistic violations at multiple levels (Hubert Lyall & Järvikivi, 2021).

Of the five traits, we were particularly interested in openness, conscientiousness, and extraversion. Openness has been attributed to better implicit learning ability (Kaufman et al., 2010) and L2 speaker’s sensitivity to frequency effects (Kerz & Wiechmann, 2017), and the same statistical learning abilities may be called on to store and utilize BTP. Conscientiousness, (though also agreeableness and neuroticism) are considered to rank how ‘sociable’ or ‘conforming’ a person is (DeYoung et al., 2002; Roberts et al., 2014), which, based on the literature, we hypothesized might lead to higher motivation in an experimental context (Stevens & Ash, 2001). Finally, extraversion may be a source of individual difference in language processing because extraverted people may be more likely to have larger or more diversified social networks, in turn making them more able to predict upcoming input, though it is unclear if this prediction could occur at the level of interlexical co-occurrence (Lev-Ari, 2019). However, we refrained from making specific or directional hypotheses on the effect of these variables, given their highly exploratory nature and lack of previous literature.

To assess these continuums, participants completed the 60-question BFI-2 inventory (Soto & John, 2017), a well-established tool in psychology, in which they were asked to self-report how much they identified with statements like “I am someone who is outgoing, sociable” (extraversion) or “I am someone who is curious about many things” (openness) on a scale of 1 to 5.

#### 1.2.5 Processing speed

The fifth and final individual difference we assess here is the overall speed with which individuals can process sensory and cognitive information, which has been long found to be a covariate of interest to individual difference studies of all types. Processing speed can be taken as an indicator of processing efficiency and the metric generally correlates with better performance on a range of higher-order psychometric tasks (Finkel et al., 2005; Kyllonen & Zu, 2016; Schubert et al., 2018; Sheppard & Vernon, 2008), although this effect may be strongly modulated by task selection (Cepeda et al., 2013).

Discussions of processing speed in linguistic research are found primarily in studies of aging and language, given that a slowing in processing speed is a well-established component of cognitive aging (Eckert, 2010; Rogalski et al., 2011; Salthouse, 1996; Soble et al., 2016). However, processing speed correlates with picture naming, auditory lexical decision, and idiom processing in young adults as well (Hintz et al., 2020; Tilmatine et al., 2021). It has been proposed to index the ease/speed with which incoming input can be integrated into unfolding representations (Huettig & Janse, 2016). And ample time is necessary to engage in predictive processing, both in a visual world context and in the reflected EEG components (Huettig & Guerra, 2019; Wlotko & Federmeier, 2015). Taken together, this suggests that processing speed is an important candidate for individual differences in language skills more broadly. However, in light of the sparse literature on the relationship between processing speed and chunking specifically, we remained agnostic to the direction of a potential effect.

To estimate the contribution of processing speed, participants completed both a simple and a complex reaction time task. For the simple task, they were instructed to press a certain key on their keyboard every time a circle appeared on their screen. This task was intended to capture sensorimotor processing speed, but as an unavoidable consequence of online data collection also includes baseline differences in hardware and internet speeds. For the complex task, they were instructed to press one key if they saw a blue circle and press a different key if the circle was orange; this was intended to capture differences in noticing visual stimuli and directing action accordingly. There were 20 trials for each of the reaction speed tasks. For both tasks, the average response time was taken to represent the reaction speed in these two conditions.

## 2. Methods

The current experiment was a self-paced reading design in which participants read unrelated sentences one word at a time. Additionally, they completed a battery of individual difference assessments, corresponding to those outlined above, as well as a three-part assessment of reading experience (for more details see McConnell & Blumenthal-Dramé, 2021) and two additional tasks that were excluded from the analysis because they were not executed correctly. The majority of these browser-based tasks were programmed in the jsPsych framework (de Leeuw, 2015; Hilbig, 2016; Reimers & Stewart, 2015). Many of the tasks were adapted from those on *Experiment Factory*, which has a wide collection of classic psychological assessments in a browser-based format (Sochat, 2018; Sochat et al., 2016). Full code for individual difference assessments is available in the OSF repository associated with this paper: https://osf.io/zmh48/. Self-paced reading and individual difference blocks were counterbalanced over participants, and the order of individual difference tasks was randomized to prevent tasks from being overly influenced by participant fatigue.

### 2.1 Stimuli

Stimuli were full sentences that were composed of three parts: semantically neutral onsets, modifier-noun bigrams and three-word spillover regions (see Table 1 for example). Bigrams were extracted from the *Corpus of Contemporary American English* (COCA) equally across 20 bins of log bigram frequency (Davies, 2008). They were then inspected by hand and removed if they were emotionally arousing (violent, religious or sexual), specialist terms from specific domains like sports or medicine (e.g., ‘underactive thyroid’), featured a modifier that was identical in form to a verbal participle (e.g., ‘cooked crab’, ‘galloping horse’), or were either idioms or compounds (e.g., ‘botanic gardens’, ‘soy cheese’). Additionally, bigrams were disqualified if either lexeme was longer than 12 letters, contained more than one base and/or more than two derivational and inflectional morphemes (e.g., ‘biodegradable’), or included a removable prefix (e.g., ‘inexperienced’), as any of this criteria could mean that the lexeme itself was a collocation at the morphological level.

**Table 1:** Stimuli lists (A and B).


LIST	SENTENCE ONSET	CRITICAL BIGRAM	SPILLOVER

A1	Everyone had heard about the	apparent conflict	between the president and his staff

A1	Despite the	apparent failure	of the blockbuster movie, many tickets were sold

A2	Despite the	obvious conflict	between the two friends, Charlotte attended the wedding

A2	Everyone had heard about the	obvious failure	of the greatly anticipated romance novel

B1	Despite the	apparent conflict	between the two friends, Charlotte attended the wedding

B1	Everyone had heard about the	apparent failure	of the greatly anticipated romance novel

B2	Everyone had heard about the	obvious conflict	between the president and his staff

B2	Despite the	obvious failure	of the blockbuster movie, many tickets were sold


Each bigram was then matched to a partner bigram with a maximally similar log-transformed bigram frequency (+/– 0.25). This allowed us to isolate the effect of the association between the individual words. After this step, we had two items with the same first word and nearly the same log bigram frequency (e.g., “absolute silence” and “absolute control”). However, we could still not rule out that if one was read faster than the other, it was an absolute effect of the second word (“silence” vs. “control”). Thus, we added two more items to the pair with the same second word and a maximally similar first word (e.g., “total silence” and “total control”), for a total of four matched items (Edmonds, 2013). The matched items were not extracted from the corpus but invented as semantic matches, so bigram frequency was not controlled across the matched condition.

**Figure 2:**
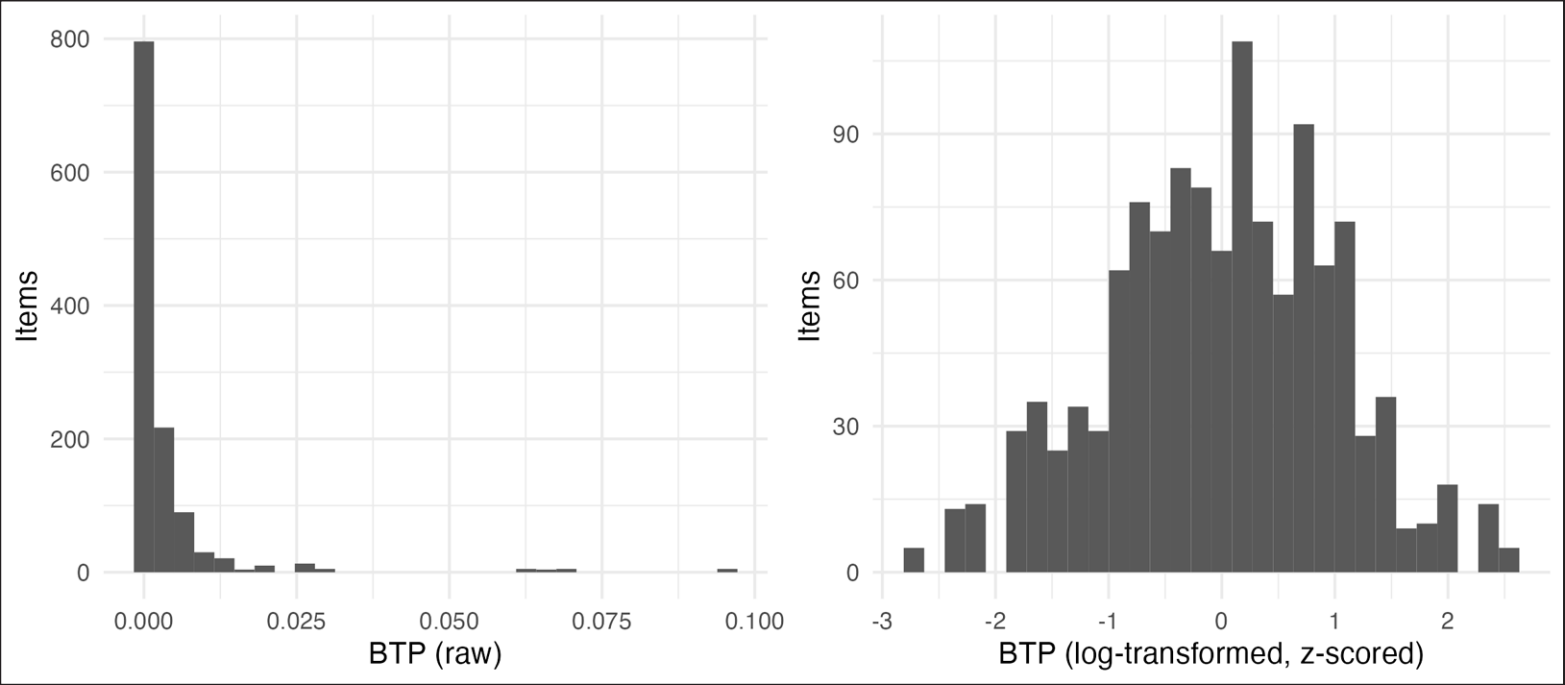
Distribution of BTP across items, raw and log-transformed.

The second word in the bigram (the noun) was designated the critical word, because this is the location in which the bigram can be fully identified, given that English noun phrases are right-headed. Log-transformed frequency of both the first word and the second word in the bigram were added to the data frame, as well as the bigram frequencies. All frequencies were extracted from COCA (2019 download version). BTP was calculated as bigram frequency divided by word 2 frequency using the same frequencies. Figure 2 shows the distribution of BTP scores across the stimuli, both in terms of raw values and the distribution after log-transformation (since the distribution is clearly Zipfian).

Items were then embedded in neutral sentences, which were counterbalanced across participants to ensure that the sentential environment did not systematically affect reading times. Table 1 exemplifies this, with participants being assigned to either List A or List B, and parts 1 and 2 being maximally spaced from each other within the course of the experiment (either all 1s before all 2s or vice versa). The same items were used in a previous experiment (McConnell & Blumenthal-Dramé, 2021).

Participants read the sentences above word-by-word in a moving window self-paced reading setup, which was based on the code by IbexFarm (Drummond, 2016). For this, participants saw lines representing the lengths of words in a sentence and were asked to press the spacebar to make the first word appear. The first word appeared over the line that corresponded to its length, and when participants pressed the spacebar again, the first word disappeared and the second word appeared. In this way, participants proceeded through all words in all sentences. Approximately one-third of sentences were followed by a comprehension question, which generally had 3 possible options (a small minority were in a yes/no format) and covered various levels of language processing from name recall to deeper syntactic understanding.

Participants read for about 45 minutes after several practice sentences and were instructed to read as normally as possible and to take breaks (e.g., to take a drink of coffee) only on the question screens. They were informed that the sentences were not related and did not form a story. After collecting data, 29 items were removed from the analysis because they were either unattested in COCA (this only affected items from the semantic match condition) or had the same first word as another item, which inadvertently happened due to the complexity of the matching process. 259 sentences per participant remained, regardless of the list they were assigned.

### 2.2 Participants

Participants were recruited online via Prolific (Damer & Bradley, 2018). Only participants who were monolingual English speakers without reading disabilities and answered 80% of SPR comprehension questions correctly were eligible; recruitment stopped when 100 such submissions were reached. Of them, 57 were female and 1 was non-binary or another gender. Ages ranged from 18 to 76 (median 30.5). 62 were UK nationals and 38 were US nationals. 46 had undergraduate education, 40 had a degree below a bachelor’s degree and 14 had a degree above a bachelor’s degree. If participants missed a task, which happened for technical reasons in a small number of cases, their score for the missing task was set to the average score across that task. This affected 4 cases for the Navon task and 2 for reading experience.

### 2.3 Individual difference measures

There was considerable variability between participants in all measures. No data was removed. Dependent variables were calculated as outlined in Table 2.

**Table 2:** Calculation of dependent variable for each individual difference assessment.


TASK	MEASURE	DEPENDENT VARIABLE

Flanker task	Cognitive inhibition	Difference between mean RT to incompatible condition and mean RT to compatible condition, over correct answers only (where higher scores denote comparatively slower responses to incompatible condition)

Navon task	Global/local scope shifting	Difference between mean RT to local condition and mean RT to global condition, over correct answers only (where higher scores denote comparatively slower responses to local condition)

Reading span task	Verbal working memory	Proportion of words recalled correctly over all trials

Big 5 (OCEAN) traits	Personality	Mean of Likert scale questionnaire (1–5) where reverse scale items were assigned negative values. Average score across all 5 subcomponents taken as 0 point (to account for individual preferences in ranking oneself) and SD calculated across all subcomponents

Complex processing speed	Processing speed	Average RT in the complex task, over correct responses only


**Figure 3:**
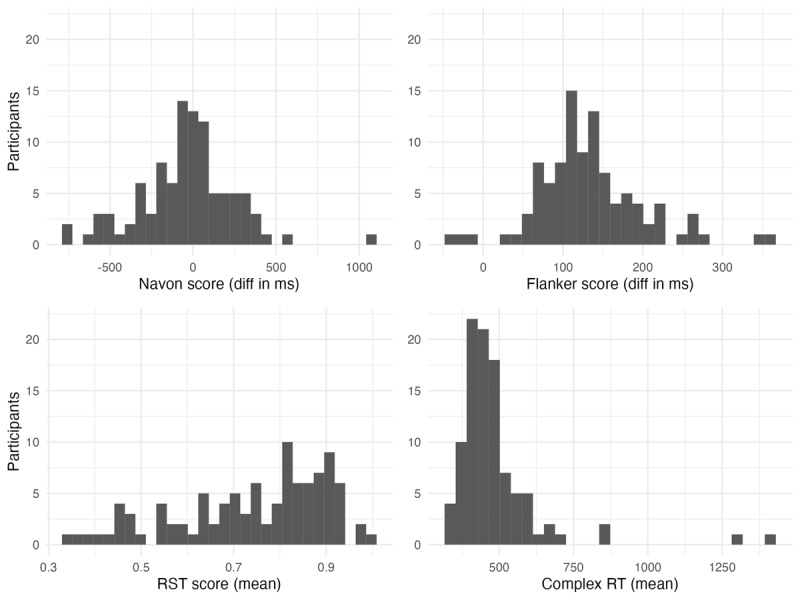
Raw scores (per participant) on the individual difference tasks.

**Figure 4:**
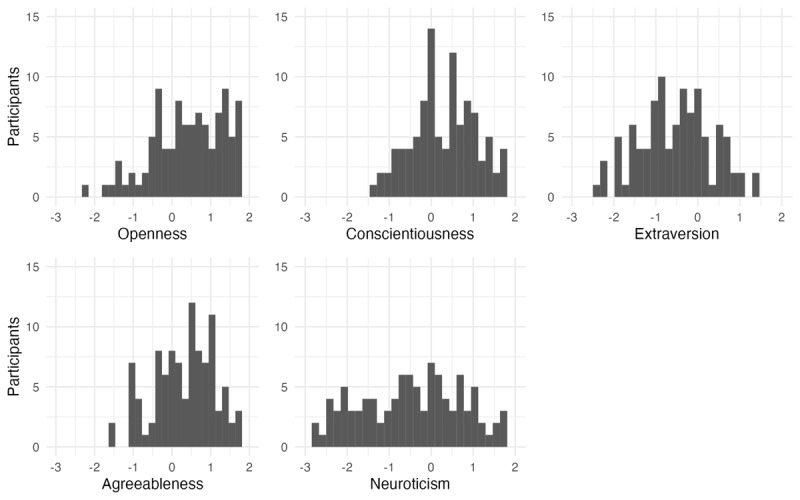
Scores on the Big Five personality components, centered at the participant’s mean score across all five components.

Figures 3 and 4 show the distribution of each of the individual difference assessments, with one value per participant. The mean, standard deviation, skewness and kurtosis of each task is also represented numerically in Table 3, along with the range of values on each task.

**Table 3:** Distribution and range of individual difference constructs, one score per participant.


CONSTRUCT	DISTRIBUTION				RANGE	
	
	MEAN	SD	SKEWNESS	KURTOSIS	MIN	MAX

Reaction speed (complex)	488.98	156.38	3.87	21.28	338.89	1416.54

Navon (global-local scope shifting)	–41.58	285.74	0.12	4.69	–767.52	1063.72

Flanker (inhibition)	133.57	67.04	0.63	4.81	–40.81	360.44

Reading span (vWM)	0.74	0.16	–0.69	2.51	0.34	1.00

Personality: Openness*	0.41	0.92	–0.52	2.64	–2.26	1.79

Personality: Conscientiousness*	0.30	0.75	–0.02	2.36	–1.30	1.79

Personality: Extraversion*	–0.54	0.86	–0.05	2.42	–2.45	1.30

Personality: Agreeableness*	0.31	0.79	–0.31	2.38	–1.59	1.79

Personality: Neuroticism*	–0.48	1.19	–0.09	2.07	–2.84	1.79


* Personality components are participant-centered (0 represents participant mean across all 5 components).

Internal consistency of each task was calculated using split-half reliability using the R package *splithalfr* (Pronk et al., 2022) using 5,000 random splits. Personality was excluded from this calculation, since the BFI-2 inventory has been independently validated (Soto & John, 2017). For the complex response time task, the Spearman-Brown corrected reliability estimate was 0.82, 95% CI [0.73, 0.91]. For the Flanker task, the estimate for the compatible condition was 0.96, 95% CI [0.93, 0.97] and for the incompatible condition was 0.94, 95% CI [0.89, 0.97]. For the Navon task, the estimate for the global condition was 0.87, 95% CI [0.82, 0.91] and for the local condition was 0.90, 95% CI [0.86, 0.93]. For the reading span task, the estimate was 0.86, 95% CI [0.81, 0.89].

### 2.4 Model specification

After data collection, the noun in the bigram was labeled the “critical word” because it is at this point that the bigram can be identified. Previous research has shown that so-called “spillover effects” can affect the reading times of subsequent words, so the two words directly following the critical word were coded as “spillover 1” and “spillover 2”, respectively. These three levels made up the “position” variable.

All numeric predictors were centered and scaled to ensure that individual difference assessments on different scales were comparable; additionally, the Big 5 personality traits were scaled to each participant’s mean response across all five tasks to account for participant differences in responding to a Likert scale (i.e., those who consistently rate themselves higher or lower on all dimensions). Response times, frequencies and transition probabilities were log-transformed (Smith & Levy, 2013). To ensure that only true reading times were included (i.e., none that were physiologically impossible or exaggeratedly long), we removed RTs outside 2.5 standard deviations from each participant’s mean (2.22%).

**Figure 5:**
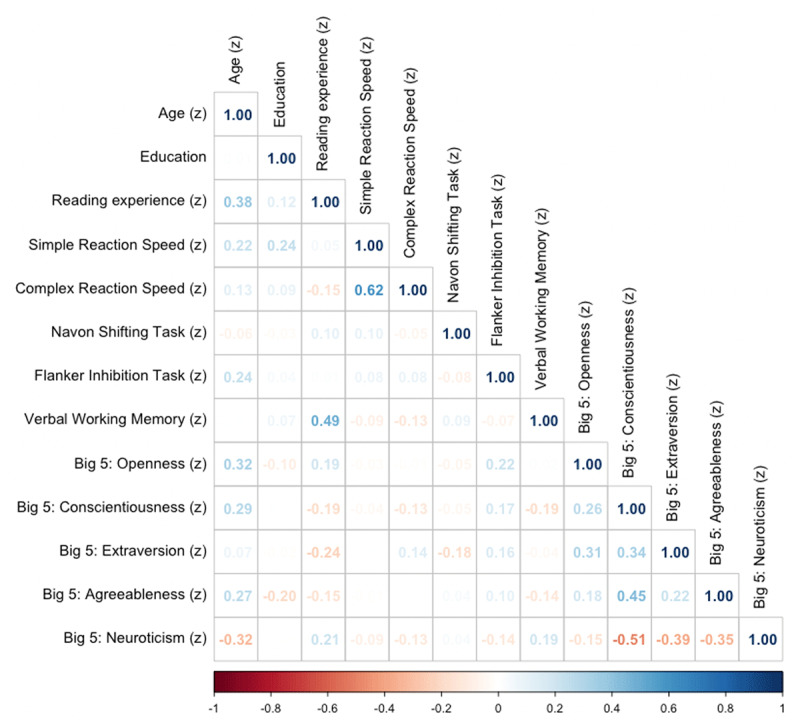
Spearman’s correlation between measures of endogenous and exogenous individual difference.

To pre-empt collinearity problems, the Spearman’s rank correlation between all individual difference assessments was consulted before model fitting (see Figure 5). If two terms were correlated above 0.4, only the one that was hypothesized to be most relevant to the research aims was included in the model. Although we collected both simple and complex reaction speeds as part of the reaction time measure, simple reaction speed was not considered in the model because it correlated strongly with complex reaction speed (ρ = 0.62) and it was deemed inferior in measuring cognitive processing speed because of how heavily it may rely on internet and hardware speed. Similarly, reading experience was not added to the model because it was strongly correlated with verbal working memory (ρ = 0.49), given that the focus of the current paper is on endogenous factors, and reading experience was intended primarily as a covariate. This does, however, align with proposals that verbal working memory may at least in part reflect language experience (Kidd et al., 2018; MacDonald & Christiansen, 2002). The exception to this threshold were individual subcomponents of the Big 5 personality traits, since these are well established. After model fitting, VIF was checked and confirmed that there were no multicollinearity problems.

The model formula included ten individual difference terms: complex response time, verbal working memory (“rst_mem”), inhibition (“flanker”), global/local shifting (“navon”), the five Big 5 personality traits, and age. Additionally, the model included the following known covariates: current word length, previous word length, position (word number) in sentence, position in experiment, place of origin (US or UK) and education level (high school or trade school, undergraduate and graduate education). Word length was used as a proxy for word frequency because word frequency is directly implicated in the calculation of BTP, the two measures are (negatively) correlated (ρ = –0.45), and previous research made the same methodological decision (McConnell & Blumenthal-Dramé, 2021).

All categorical predictors were sum coded. A linear mixed effects model was fit in Julia using the package *MixedModels.jl* (Bates et al., 2021) and *JellyMe4.jl* (Alday, 2023), and plotted in R using *lme4* (Bates et al., 2015) and *ggeffects* (Lüdecke, 2018). Random effects were fit on three grouping variables: participant ID, first word in the bigram, and second word in the bigram. Due to the stimuli design, this controlled for the paired quads that were designed to minimize the effects of individual words. Random slopes were fit to word-level covariates. After model fitting, the principal components of the random effects were checked for overparameterization, but no problems were detected, so the random effects structure did not need to be edited.

A single step of forward model selection was performed after finalizing the random effects structure, in which a three-way interaction term by position was added only for those individual difference terms that showed significant two-way interactions with BTP. This was intended to account for hypotheses about the time course of effects while minimizing the number of three-way interactions that the model included.

The final model formula is below, written in Julia model syntax conventions (@formula)^2^.

logRT ~ 1 + BTP_lz & navon_z & position + BTP_lz & flanker_z & position + BTP_lz * (complex_rt_z + rst_mem_z + navon_z + flanker_z + big5_O_cz + big5_C_cz + big5_E_cz + big5_A_cz + big5_N_cz + age_z) + position + trial_number_z + word_number_z + length_z + prev_length_z + origin + education +

(1 + trial_number_z + word_number_z + length_z + prev_length_z | id) +

(1 + trial_number_z | w1) +

(1 + trial_number_z| w2));

After model fitting, model diagnostic plots and assumptions were checked and no violations were detected. No VIF scores were above 2. The full analysis code is available in the OSF repository associated with this paper: https://osf.io/zmh48/.

## 3. Results

**Figure 6:**
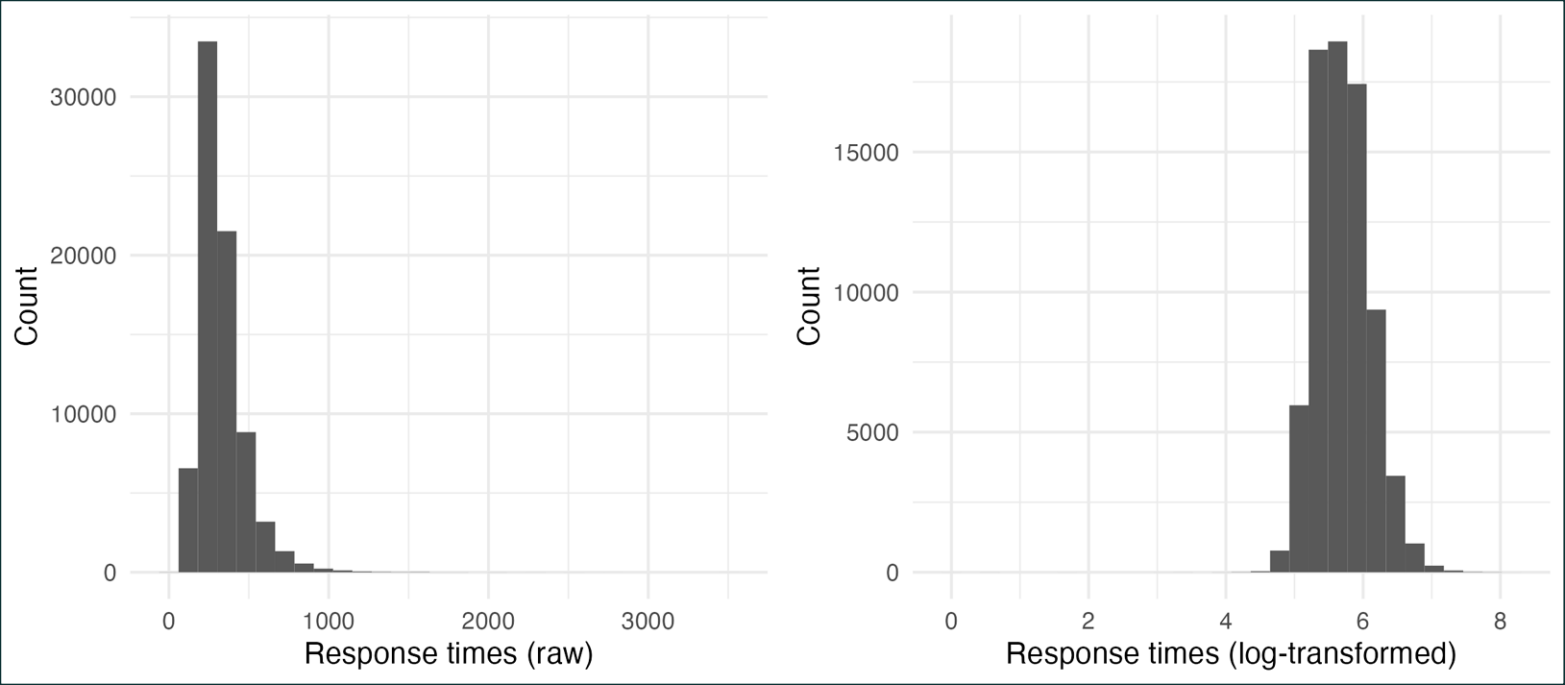
Distribution of response times, raw and log-transformed.

Response times were log-transformed. Figure 6 shows the distribution of the RTs before and after log transformation.

Table 4 shows the full model output. The reported p-values are from the *MixedModels.jl* package, which uses the infinite degrees of freedom approximation in transforming Wald statistics to z-values (Baayen et al., 2008; Bates et al., 2021). All covariates show the expected effect: progressing toward the end of a sentence, as well as progressing through the experiment lead to a quickening effect on response times (β = –0.0058, SE = 0.0023, p = 0.0132; and β = –0.1139, SE = 0.0063, p < .0001, respectively). Longer words and longer preceding words lead to slower response times (β = 0.0122, SE = 0.0026, p < .0001; and β = 0.0266, SE = 0.0030, p < .0001, respectively). The critical word (the noun in the bigram) was read overall more quickly (β = –0.0087, SE = 0.0020, p < .0001) and the word directly after it (the first spillover word) was read more slowly (β = 0.0149, SE = 0.0014, p < .0001) when compared to the grand mean over the three positions (noun, first spillover word, and second spillover word).

**Table 4:** Output of linear mixed effects model.


LINEAR MIXED MODEL FIT BY MAXIMUM LIKELIHOOD							

Variance components:							

	**Column**	**Variance**	**Std.Dev**.	**Corr**.			

Id	(Intercept)	0.0683	0.2614				
	trial_number_z	0.0038	0.0618	–0.12			
	word_number_z	0.0004	0.0189	–0.18	–0.14		
	length_z	0.0005	0.0232	0.63	–0.02	–0.36	
	prev_length_z	0.0007	0.0263	0.57	0.08	–0.32	0.49
w2	(Intercept)	0.0002	0.0140				
	trial_number_z	0.0001	0.0094	–0.30			
w1	(Intercept)	0.0003	0.0185				
	trial_number_z	0.0001	0.0077	0.05			
Residual		0.0597	0.2444				
Number of obs: 75973; levels of grouping factors: 100, 143, 135							

Fixed-effects parameters:							

	**Coef**.	**Std. Error**	**z**	**Pr(>|z|)**			

(Intercept)	5.7076	0.0289	197.8000	<.0001			
**BTP_lz**	**–0.0051**	**0.0017**	**–2.9400**	**0.0033**			
complex_rt_z	0.0251	0.0224	1.1200	0.2617			
rst_mem_z	0.0423	0.0215	1.9700	0.0490			
navon_z	–0.0024	0.0203	–0.1200	0.9053			
flanker_z	0.0100	0.0212	0.4700	0.6355			
big5_O_cz	–0.0116	0.0228	–0.5100	0.6106			
big5_C_cz	0.0167	0.0254	0.6600	0.5120			
big5_E_cz	0.0064	0.0231	0.2800	0.7811			
big5_A_cz	0.0029	0.0224	0.1300	0.8984			
big5_N_cz	–0.0434	0.0259	–1.6800	0.0937			
**age_z**	**0.0467**	**0.0232**	**2.0100**	**0.0440**			
**position: noun**	**–0.0087**	**0.0020**	**–4.4300**	**<.0001**			
**position: spillover_1**	**0.0149**	**0.0014**	**10.7300**	**<.0001**			
**trial_number_z**	**–0.1139**	**0.0063**	**–17.9900**	**<.0001**			
**word_number_z**	**–0.0058**	**0.0023**	**–2.4800**	**0.0132**			
**length_z**	**0.0122**	**0.0026**	**4.6400**	**<.0001**			
**prev_length_z**	**0.0266**	**0.0030**	**8.9900**	**<.0001**			
origin: UK	0.0069	0.0223	0.3100	0.7564			
education: Grad school	0.0316	0.0373	0.8500	0.3971			
education: Undergraduate	–0.0210	0.0281	–0.7400	0.4563			
BTP_lz & complex_rt_z	–0.0016	0.0011	–1.4900	0.1371			
BTP_lz & rst_mem_z	0.0000	0.0010	0.0300	0.9738			
**BTP_lz & navon_z**	**0.0022**	**0.0010**	**2.2200**	**0.0265**			
**BTP_lz & flanker_z**	**0.0023**	**0.0010**	**2.2200**	**0.0265**			
BTP_lz & big5_O_cz	0.0006	0.0011	0.5600	0.5774			
BTP_lz & big5_C_cz	–0.0021	0.0012	–1.6800	0.0930			
BTP_lz & big5_E_cz	0.0002	0.0011	0.1600	0.8748			
BTP_lz & big5_A_cz	0.0020	0.0011	1.8900	0.0592			
BTP_lz & big5_N_cz	0.0003	0.0012	0.2600	0.7949			
BTP_lz & age_z	–0.0014	0.0011	–1.2400	0.2140			
BTP_lz & navon_z & position: noun	0.0001	0.0013	0.0700	0.9476			
BTP_lz & navon_z & position: spillover_1	–0.0001	0.0013	–0.0700	0.9415			
BTP_lz & flanker_z & position: noun	0.0011	0.0013	0.8900	0.3750			
BTP_lz & flanker_z & position: spillover_1	–0.0018	0.0013	–1.4300	0.1534			


Backward transition probability (BTP) also affected response times, so that items with higher BTP were read more quickly (β = –0.0051, SE = 0.0017, p = 0.0033). Higher verbal working memory in terms of the reading span task seems to correlate with slower response times in general, but this effect is not statistically significant (see recommendation in Baayen et al., 2008 to interpret z-scores of >2 as corresponding to an alpha level of 0.05). Similarly, older age correlated with slower response times (β = 0.0467, SE = 0.0232, p = 0.0440) but the interaction with BTP is not statistically significant in the current model.

**Figure 7:**
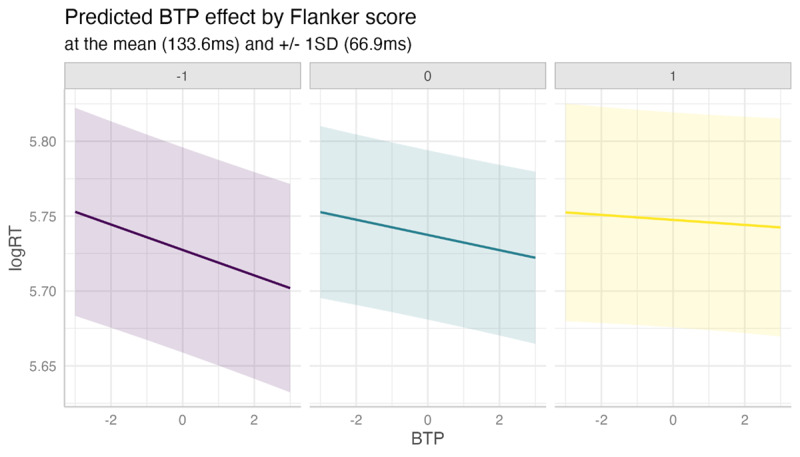
Interaction between (centered, z-scored) backward transition probability and (centered, z-scored) Flanker score, in which higher score represents a greater slowing in the incompatible distractor condition.

**Figure 8:**
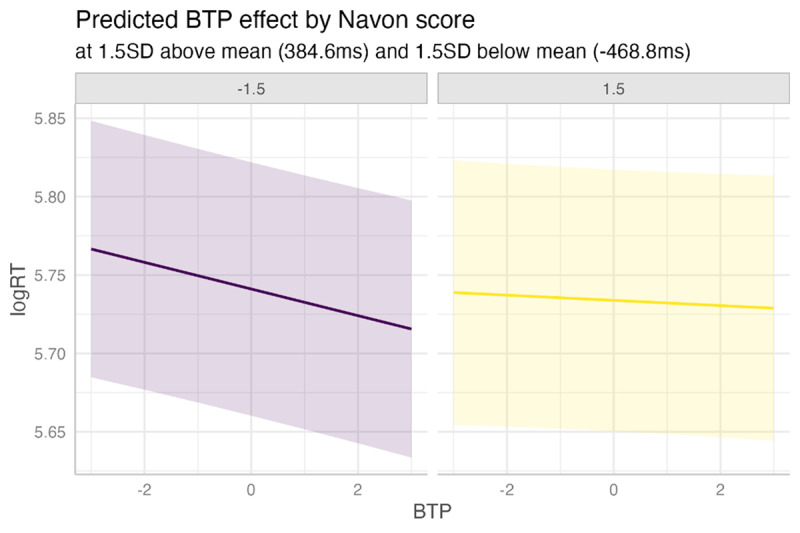
Interaction between (centered, z-scored) backward transition probability and (centered, z-scored) Navon score, in which higher score represents overall slower responses in the local condition (and lower score shows overall slower responses in the global condition).

Two individual differences show significant interactions with BTP: those corresponding to the Navon global-local shifting task (β = 0.0022, SE = 0.0010, p = 0.0265) and to the Flanker inhibition task (β = 0.0023, SE = 0.0010, p = 0.0265). These two effects are of a very similar size although they are not strongly correlated (ρ = 0.08). Figure 7 shows a marginal effects plot of the Flanker—BTP interaction effect at three conceptually important levels: the mean score difference between the compatible and incompatible conditions (133.6 ms) and at 1 SD (66.9 ms) above and below the mean (i.e., an average speed penalty of 200.5 ms and 66.7 ms for the incompatible compared to the compatible condition, respectively). Similarly, Figure 8 shows the Navon—BTP interaction at two conceptually important levels: 1.5 SD above the mean (384.6 ms slower in the local condition) and 1.5 SD below the mean (468.8 ms faster in the local condition).

No evidence was found for a modulating effect of complex reaction time, verbal working memory, or personality on BTP effects. Similarly, we did not find evidence that three-way interactions with position, BTP, and either Navon or Flanker were supported by the data. Note, however, that mixed effects models generally need a great deal of data to find significant three- and even two-way interactions (Heo & Leon, 2010); thus we simply fail to reject the null hypothesis here and do not claim that there are no real effects at the population level.

## 4. Discussion

In the current experiment, we sought to correlate measures of endogenous individual difference with the interlexical statistical association measure of backward transition probability (BTP). Results of the self-paced reading task show that those modifier-noun bigrams with a high BTP score, i.e., those that have a high chunk frequency compared to frequency of the head noun, were read more quickly than bigrams with a lower BTP score. This held when controlling for well-established self-paced reading covariates like word length, previous word length, word number in sentence and position in the experiment. Raw reading times were also modulated by age, with older participants reading more slowly, and by verbal working memory, with participants scoring higher also reading more slowly.

The effect is found across all three words in the critical region: the noun which signifies the point where the bigram can be identified and integrated, as well as the two words following it. The design thus allows for spillover effects to be captured, although there is no statistically significant three-way interaction with BTP and any of the significant individual difference assessments, so we cannot confirm hypotheses about the time course of effects (Smith & Levy, 2013). However, support for three-way interactions with a three-level categorical variable in a mixed effects model demands considerable statistical power, so it is possible that the effect size is simply too small to be captured by the current dataset, and the question remains interesting for future research.

To draw conclusions about which endogenous factors affect a reader’s reliance on interlexical statistics, however, we must compare the interacting effects of BTP and individual difference factors. Interestingly, three of the five sources of individual difference do not show significant interactions with BTP. Verbal working memory ability does not affect how readily or efficiently readers can access word co-occurrence relationships, or the likelihood with which they access these at all (contrasted with storing and retrieving chunked multi-word units). This is particularly interesting in light of previous research that claims that verbal working memory is implicated in combinatorial mechanisms of prediction involving linking language input to visual locations (Huettig & Janse, 2016) and that working memory is tied to relevant abilities like statistical learning (Medimorec et al., 2019). It should be noted, however, that our administration of the reading span task was participant-administered, which may not make it comparable to previous research that used in-lab researcher-administrated versions of the task (Friedman & Miyake, 2004a); participants may not have read sentences out loud despite being asked to, because this significantly impairs memory retention. Thus, replicating the experiment in a lab setting would be desirable.

Similarly, the results do not show an effect of overall processing speed. Processing speed, however, has been found to be affected by task, and word-by-word reading may have impeded some strategies that would otherwise be present in naturalistic reading (Cepeda et al., 2013). Additionally, our measure of processing speed was based on a small number of trials and participants responded on personal computers and internet connections that may have varied widely. Finally, personality as defined by the Big Five characteristic set also did not show interaction effects with BTP, although two of the traits may warrant future research (Conscientiousness and Agreeableness). Personality as an individual difference construct is still relatively under-researched despite recent interest (Hubert Lyall & Järvikivi, 2021), so it is difficult to postulate why the traits did not appear to affect this aspect of language processing, although some have been correlated with implicit learning and sensitivity to frequency (Kaufman et al., 2010; Kerz & Wiechmann, 2017).

Two individual difference assessments interact significantly with BTP in predicting self-paced reading response times and they both involve dynamics between parts and wholes: the cognitive inhibition ‘Flanker’ assessment and the global-local scope ‘Navon’ task. Both of these tasks reflect a relationship between the part and the whole: In the Flanker task, participants must focus on the part in the middle of the displayed figures, while inhibiting distracting information from the global environment. The score is the processing time delay when the global environment is distracting. In the Navon task, the score reflects the difference in timing between a participant’s ability to answer on the local level and at the holistic level. Thus, larger values in the Flanker task correspond to stronger distraction by stimuli in the global environment, and larger values in the Navon task represent a speed advantage for the global processing condition. In both assessments, higher scores represent readers adopting a more strongly holistic strategy to processing; those with higher scores show more reliance on larger units compared to the parts in isolation.

Of course, both of these tasks involve purposeful and goal-directed attention to either the global or the local level, which need not be correlated with naturalistic reading of bigrams that could be understood either holistically or compositionally. Interestingly, both of these abilities show a similar pattern in their interaction with BTP; those participants who scored higher on either the Flanker or the Navon task show reduced effects of BTP on response times. Because a high score on either of these tasks points to a stronger interference of or easier access to the holistic level, it seems for these participants, the cognitive precedence of the whole is stronger when compared to other participants. This holistic bias thus also affects reading even when attention is not specifically directed to the holistic level; the association strength between individual words, and thus effect of the interlexical statistical measures like BTP, is reduced in participants who are more inclined to processing at the holistic level.

We assume that processors who proceed in a more holistic manner engage less often in computation of relationships between parts; that is to say, computing associations based on statistical co-occurrence is not their primary processing level. However, given that the scores on the two tasks do not measure the same underlying construct and are not correlated (ρ = –0.08), what difference is there between the participants who scored highly on each task? The Flanker task always asks participants to attend to a smaller grain size, without switching between levels, and the score represents the participant’s ability to do this without being distracted by irrelevant stimuli. Thus, it is a conscious ability to exert control and monitor distraction and doesn’t require any task switching between trials. It may be effortful to inhibit the global environment if it is strongly activated. The Navon task, on the other hand, asks participants to flexibly switch between the global and the local scope trial-by-trial. Thus, they are asked to attend to both levels and quickly switch attention. Because this must happen so spontaneously, the scores more likely represent a default preference or an automatic, unconscious bias. It is reasonable to anticipate that some participants may have a quick, unconscious processing bias for the part level, and thus a low Navon score, but have difficulty fully inhibiting the whole and focusing exclusively on a part, thus a high Flanker score, and vice versa.

The relevance of these two uncorrelated yet conceptually similar tasks suggests that there may be multiple paths to the same outcome amongst those individuals who perform best at the holistic level: either the chunk is immediately activated, becoming available sooner than the individual units (as would seem to align with a high score in the Navon task), or the holistic level may be attended to later and more consciously, but also more strongly (as would seem to align with a high score in the Flanker task). The analysis suggests that both of these pathways may be sources of individual difference. However, neither seems to affect reading times in general, as there is no main effect of either task on response time, and there is no three-way interaction with position, which would suggest a differential time course in the spillover region.

This conclusion suggests that individuals may differ both in their abilities and in their pathways to the same goal, which has implications for theories of holistic processing. Take for example the ongoing question of whether chunking effects are a result of a fusing of units at smaller grain sizes into a larger unit that is immediately accessed instead of its component parts, or whether individual parts in high frequency units can simply be more quickly retrieved and assembled (Blumenthal-Dramé, 2017; Blumenthal-Dramé et al., 2017; Carrol & Conklin, 2020; Conklin & Schmitt, 2012; Lorenz & Tizón-Couto, 2019; Siyanova-Chanturia, 2015; Siyanova-Chanturia et al., 2011, 2017). The answer to this question may remain elusive because it cannot be answered in a categorical way, but rather depends on individual differences in processing style. Some individuals may be quick and efficient in bottom-up processing and thus rely more heavily on interlexical statistics (exemplified here as BTP), whereas others retrieve the whole either more quickly or more strongly, and thus the whole is more cognitively prominent to them.

In general, we find that those individuals for whom the holistic level is more prominent rely less on interlexical statistics, such as those captured by BTP. However, this group is not homogenous; some seem to have the holistic level as a default processing depth whereas others may access the holistic level later, but more strongly than the component parts. Thus, a higher co-occurrence between individual words may not assist those readers who proceed at a holistic level. An open question remains on whether holistic processing is more efficient than compositional processing in general. That is, are they two paths to the same goal? Or is one strategy more viable than the other, leading to quicker and more efficient language processing?

Although we did not find any evidence that one of these pathways is more efficient, there may be task-based reasons for this. Reading words one-by-one in a moving window format might disadvantage those readers who naturally process at larger grain sizes. Perhaps the experimental presentation disfavored holistic processors particularly; follow-up research involving multi-word self-paced reading presentation is planned on this question.

Future research is also necessary into global/local scope shifting on different levels. When we talk about the Navon task as revealing a preference either for the “global” or the “local” scope, this binarizes what might not be a clear-cut categorical distinction, and which may not map entirely onto the levels of a bigram and its component words. The individual words that make up the bigram could be further decomposed into component morphemes, which could lead to the conclusion that the individual words themselves are not the “local” level but rather an intermediate level (Yang et al., 2022, 2020). Indeed, research shows that co-occurrence on the morphemic level informs processing of multimorphemic words (Blumenthal-Dramé et al., 2017). The findings of the current paper would suggest that the shifting ability utilized in a Navon task maps to reading multi-word units, but it would be interesting to pursue if this holds on other levels of detail.

It is also important to recognize that the current results only show that individual differences in global/local scope shifting and inhibition affect reliance on BTP at the level of lexical co-occurrence between modifiers and nouns. Future research should replicate the current findings in different stimuli sets, where holistic and compositional processing are not limited to modifier-noun bigrams in particular. For example, do these biases also show up on the phonological or syntactic level? Does BTP affect reading times in all regions of the sentence? And do the same individual differences underlie processing in other areas, or do other individual difference factors arise as important? Assessing these abilities in language acquisition would also be of interest. Are differences between holistic and compositional processing styles visible in learners and does this lead to differences in achievement, or is there a mediating factor that correlates with both outcome and ability? Finally, the effect of statistical learning ability, which has been found to correlate with some of the abilities we tested, should also be directly assessed as a predictor of bigram reading (Isbilen et al., 2022).

## Data Accessibility Statements

The raw data supporting the conclusion of this article will be made available by the author, without undue reservation. Analysis code and experimental specifications are available in an online repository: https://osf.io/zmh48/.

## Footnotes

Ex: Given the item “> > < > >”, participants should respond with the key corresponding to left (here: “F”) but given “> > > > >”, they should respond with the key corresponding to right (here: “J”).

According to this syntax, 1 represents the grand intercept, + lists all main effects, and & represents an interaction term and the accompanying main effects. Random effects terms are in rounded parentheses, where 1 represents a random intercept and + lists the random slopes, by the grouping variables that are denoted with |.
